# Extremely High Thermal Conductivity of Aligned Carbon Nanotube-Polyethylene Composites

**DOI:** 10.1038/srep16543

**Published:** 2015-11-10

**Authors:** Quanwen Liao, Zhichun Liu, Wei Liu, Chengcheng Deng, Nuo Yang

**Affiliations:** 1 School of Energy and Power Engineering, Huazhong University of Science and Technology (HUST), Wuhan 430074, China; 2 Nano Interface Center for Energy (NICE), School of Energy and Power Engineering, Huazhong University of Science and Technology (HUST), Wuhan 430074, China; 3 State Key Laboratory of Coal Combustion, Huazhong University of Science and Technology (HUST), Wuhan 430074, China

## Abstract

The ultra-low thermal conductivity of bulk polymers may be enhanced by combining them with high thermal conductivity materials such as carbon nanotubes. Different from random doping, we find that the aligned carbon nanotube-polyethylene composites has a high thermal conductivity by non-equilibrium molecular dynamics simulations. The analyses indicate that the aligned composite not only take advantage of the high thermal conduction of carbon nanotubes, but enhance thermal conduction of polyethylene chains.

Polymers have been widely utilized in all walks of life due to their outstanding physical properties, such as high toughness, low density, and corrosion resistance. However, its poor heat transfer ability limits its applications^1^,^2^. The ultra-low thermal conductivities (κ), on the order of 0.1 Wm^−1^ K^−1^ at room temperature^3^, comes from the structures of random and twisting chains which cause a lot of phonon scatterings and leads to a short phonon mean free path.

Recently, it is reported that a suspended polymer chain and oriented polymer chains have remarkable thermal conductivities^4^,^5^,^6^,^7^,^8^,^9^. Chen’s group predicted that the thermal conductivity of a suspended polyethylene chain (SPEC), κ_SPEC_, achieved as high as 350 Wm^−1^ K^−1^ at room temperature by molecular dynamics (MD) simulation^4^,^5^. Moreover, they measured the thermal conductivity of ultra-drawn polyethylene (PE) nanofibers, κ_PE_, as 104 Wm^−1^ K^−1^ using the cantilever method^6^. Besides, Virendra *et al.* measured the κ of amorphous polythiophene nanofibers as 4.4 Wm^−1^ K^−1^ at room-temperature and calculated κ as 43.3 Wm^−1^ K^−1^ by MD for a suspended polythiophene chain^7^. Moreover, Zhang *et al.* demonstrated that a high thermal conductivity and good stability could be achieved in polymers with rigid backbones^2^. However, it is quite difficult to take advantage of a single polymer chain in bulk structures.

Another way to enhance thermal property of polymer structure is to produce polymer/carbon nanotube composites. The carbon nanotube (CNT) has been well studied since its discovery in 1991^10^,^11^. It is found that CNT has a super-high thermal conductivity on the order of 1000 Wm^−1^ K^−1^ at room temperature^12^. Some efforts have been made in fabricating polymer/CNT composites which have both a better thermal transport properties than bulk polymer^13^,^14^,^15^,^16^,^17^,^18^,^19^,^20^,^21^,^22^,^23^,^24^,^25^. However, the interfacial thermal resistance between CNT and polymer obstructs a further enhancement in thermal transport^23^,^26^,^27^. It was suggested that a strong coupling between CNTs and polymers could reduce phonon scatterings at interfaces and effectively improve the thermal transport in composites^26^,^27^.

In order to further enhance thermal properties of PE composites, we investigated numerically the thermal conductivity of aligned carbon nanotube-polyethylene composites (ACPCs) in this paper (structure shown in Fig. 1 (a)). Here, the well-studied (10, 10) single-walled carbon nanotube (SWCNT) and PE chains are chosen. The ACPC structure is based on a SWCNT array system, and PE chains are aligned with SWCNTs, which will avoid both disordered and interfacial phonon scatterings existed in amorphous PE composites. In the following, we show firstly a description of the model and simulation procedures. Secondly, we discuss simulation results and analyze mechanism. The results show that the non-bonded interactions between the parallel-aligned SWCNT and PE chains could enhance significantly the thermal conductivity of PE chains. Our study may inspire productions and measurements of aligned carbon nanotube and polymer-based composites.

## Results and Discussions

The main results are shown in Fig. 2 which includes the thermal conductivities of a suspended SWCNT, a SPEC, and several ACPCs with different structures. The value of κ_SWCNT_ reaches 155 Wm^−1^ K^−1^ when the length of the SWCNT is 160 nm. Obviously, the κ of (10, 10) SWCNT is not converged and will keep diverging as its length increases. Our result is slightly smaller than previous reported simulation results^28^,^29^,^30^,^31^, due to the difference of empirical potential. The simplified Morse potential neglects some interactions within the SWCNT, such as dihedral and van der Waals interactions. That is, our result is conservative and undervalue the κ_SWCNT_ a little.

Similarly, the thermal conductivity of a SPEC also shows a strong length dependence. The κ_SPEC_ achieves 57 Wm^−1^ K^−1^ with a length of 160 nm at room temperature. Compared with previous simulation results, our result is less than the Hu’s^2^, 104 Wm^−1^ K^−1^ at 160 nm length, and a little higher than the Zhang’s^32^, 49 Wm^−1^ K^−1^ with at 50 nm length. The discrepancy between them is chiefly derived from the different models used for the PE chain. Such as, a simplified model of a PE chain is applied in Hu’s simulations, where methylene (CH_2_) groups are regarded as united atoms. Moreover, in Zhang’s work, a different potential (COMPASS) is used to model the PE chain. As a SPEC possesses a much higher thermal conductivity than that of an amorphous bulk PE, we will take advantage of this property in enhancing the κ of PE-based materials.

The most significant finding is that the thermal conductivities of ACPCs are not only three orders higher than the bulk PE, but almost twice as large as a SPEC. That is, the κ of PE composite is greatly enhanced by the SWCNT’s reinforcement. In our simulations, the maximum value of κ_ACPC_ is 99.5 Wm^−1^ K^−1^ for an ACPC 3–8 with a length of 320 nm, which is comparable to that of measurements in ultra-drawn PE nanofibers, around 104 Wm^−1^ K^−1^ 6. Moreover, the κ_ACPC_ is just limited by the simulation cell’s length and could reach a much more higher value with the increasing of length due to the divergence behavior of κ in low dimensional structures^33^. Furthermore, there are few reports on polymer composites with such a high thermal conductivity and the κ_ACPC_ is at least 30 times higher than the reported κ of CNT-polymer composites^22^,^24^,^25^. As mentioned in the introduction, in the work by Virendra *et al.*^7^ the measured κ of chain-oriented amorphous polythiophene nanofibers is about one-tenth of the MD calculated κ of a suspended polythiophene chain at room-temperature. Besides, in the work by Chen’s group^4^,^5^,^6^, the measured κ of ultra-drawn PE nanofibers is about one-third of the MD calculated κ of SPEC. Thus, estimated by previous works, we would predict that the measured κ of the ACPCs is in a scope from 10 to 33 Wm^−1^ K^−1^ based on current fabricating technologies.

The high thermal conductivity of ACPC attributes to three mechanisms. Firstly, the SWCNTs possess a high thermal conductivity, which contributes a lot in enhancing the thermal conductivity of the PE-based composites. Secondly, instead of random doping, the SWCNTs are aligned with PE chains, which is the most important factor. The aligned structures not only take advantage of the divergent κ of PE chains with length, but avoid the interface scattering issue between SWCNTs and PE chains in composites. Thirdly, it was found that the non-bonded interactions between the SWCNTs and PE chains also have a significantly positive effect on the thermal transport in ACPCs. The van der Waals forces between the SWCNTs and PE chains hinder vibrations, inducing a crystal-like structure in the PE chains. Hence, the thermal conductivity of the PE chains within an ACPC is improved by the SWCNT interactions to become even higher than that of a SPEC.

Besides the length dependence, the thermal conductivity of ACPC also depends on the number of chains inside SWCNTs, M (shown in Fig. 2). As the number M increases, the κ_ACPC_ first increases and then decreases. A maximum value of thermal conductivity was observed when there are three PE chains inside SWCNTs. Due to the space limitation inside a SWCNT, the van der Waals interactions increase with an increasing number of PE chains within a SWCNT. The van der Waals interactions could take two competitive effects. When 3 or fewer chains are placed inside the SWCNT, there is a slight van der Waals interaction which can suppress the transversal bending of chains and enhance the heat transfer. However, when M is above 3, stronger interactions will bring more phonon scatterings which decrease the thermal conductivity.

In the following, we show a further analysis of the mechanism in the thermal conductivity enhancement of PE chains within an ACPC. As shown in superimposed images (inserts of Fig. 3), the PE chains within ACPC 3–12 have a clear crystalline structure compared with the SPEC. Accordingly, shown in Fig. 3, the radius density profile, g(r), of a SPEC appears amorphous, suggesting a large spread of atom vibrations and many segmental rotations of chain. In contrast, the g(r) of a PE chain within ACPC 3–12 has clear peaks and valleys, corresponding to a more ordered crystal lattice. That is, the van der Waals forces in ACPC make PE chains crystal-like and reduce disorder phonon scatterings survived in suspended chain.

We keep analyzing the details of the enhancement of thermal conductivity by ACPCs. In Fig. 4(a), it shows that the thermal conductivity of 20 nm ACPC 3-N changes a little on No. of PE chains outside the SWCNT (N). A maximum value of 63.7 Wm^−1^ K^−1^ was obtained for the ACPC 3-N thermal conductivity when N is 8. Besides, we pick up the heat flux (J) of PE chains alone in ACPCs. The thermal conductance is defined as G = *κ*·A/*L* = −*J*/Δ*T*, where *κ*, *A*, and *L* are the thermal conductivity, cross-section area, and length, respectively. The thermal conductance of PE chains in ACPCs (the blue circles) is compared with that of a 20 nm SPEC (blue dashed line) shown in Fig. 4(a). It shows that there is a significant enhancement in the thermal conductance of PE chains in the ACPCs. The G_PE_ of ACPC 3–4 is around four times larger than G of SPEC, since the non-bonding interactions in ACPC make a more crystal-like PE structure. That is, the high *κ* of ACPC comes from not only SWCNT but the PE chains.

Fig. 4(b) shows the thermal conductivity of ACPCs versus PE content for ACPC M-12 and 3-N structures. With increasing PE content, the thermal conductivity of ACPCs does not decrease monotonically, although κ_PE_ is much smaller than κ_SWCNT_. For example, the thermal conductivity of 20 nm ACPC 3-N is not sensitive to the increase of PE content. For the 20 nm and 40 nm ACPC M-12, an increase is observed when the PE content increase from 19.5% to 22%. Therefore, the PE chains in ACPCs do account for a significant contribution in thermal transport.

Moreover, Fig. 4(c) shows the contribution of PE chains and SWCNT to thermal conductance of ACPC 3–8 and ACPC 3–4 with 80 nm in length. It shows that the PE chains contribute a considerable percentage of the total thermal transport, 36.4% (27.8%) for ACPC 3–8 (3–4). Both the G values of SWCNTs in ACPC 3–4 (22.6 × 10^−10 ^WK^−1^) and ACPC 3–8 (23.86 × 10^−10^ WK^−1^) are smaller than that of a suspended 80 nm SWCNT (26.32 × 10^−10^ WK^−1^) due to the scattering from non-bonding interactions in ACPC. The values of thermal conductance per PE chain inside and outside the SWCNT in ACPC 3–8 and 3–4 are shown in Fig. 4(d). Compared with an 80 nm SPEC, the G value per PE chain is improved by as large as 38.5% for chains inside SWCNT in ACPC 3–8. That is, the non-bonded interactions in ACPCs enhance the thermal transport of the PE considerably, about 23% on average.

## Conclusions

We propose a new composite model as aligned carbon nanotube-polyethylene composites, namely ACPCs. The thermal conductivity (κ) of ACPCs are studied by non-equilibrium molecular dynamics simulations. The most significant finding is that the thermal conductivities of ACPCs are not only three orders higher than the bulk PE, but almost twice as large as a suspended PE chain which is well known by its high κ. The κ_ACPC_ is also at least 30 times higher than the κ of other reported CNT-polymer composites. Besides, there is a large enhancement (~23%) of thermal conduction for PE chains in ACPCs even comparing with a suspended PE chain. So that, the PE chains have a considerable contribution (~30%) to the thermal transport in ACPCs.

The high thermal conductivity of ACPCs attributes to the high thermal conductivity SWCNTs, the aligned SWCNTs with PE chains, and the non-bonded interactions between SWCNTs and PE chains. Our predictions may inspire manufacturing aligned polymer-based composites for a wide variety of applications.

## Methods

Classical non-equilibrium molecular dynamics (NEMD) simulations are used to study the thermal conductivity of the SWCNT, the SPEC and the ACPC. All simulations are performed by the large-scale atomic/molecular massively parallel simulator (LAMMPS) package^34^. The temperatures of the heat source and heat sink are set at 310 K and 290 K, respectively. The fixed boundary condition is applied in the longitudinal direction and the periodic boundary conditions are applied in the two transversal directions. The cross-section of ACPC simulation cell after relaxation spreads from 400 to 500 Å^2^ which depends on the structure of ACPC.

The potential energy of the SWCNT is described by a Morse bond and a harmonic cosine angle for bonding interactions, which includes both the two-body and three-body potential terms^35^,^36^,^37^. The atomic interactions of PE chains are described by an adaptive intermolecular reactive empirical bond order (AIREBO) potential^38^, which is developed from the second-generation Brenner potential^39^. In addition, the non-bonded interactions between the SWCNT and PE are described by the Lennard-Jones potential:


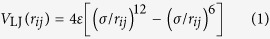


where 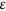 is the depth of the potential well, 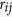 is the distance between atom i and j. The Lennard-Jones parameters are σ_SC−PC_ = 3.

Å, 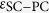 = 0.0028 eV, 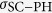 = 3.025 Å, and 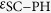 = 0.0021 eV; the SC, PC, and PH subscripts represent the carbon atoms within the SWCNT, the carbon atoms within the PE chains, and the hydrogen atoms within the PE chains, respectively. Additionally, an 8.5 Å cutoff distance is used for the 12–6 Lennard-Jones interaction.

**Figure 1:**
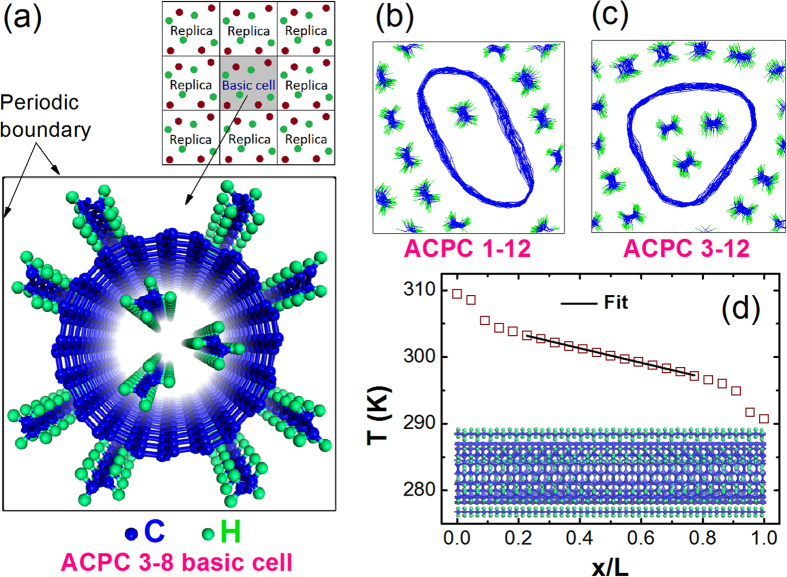
Schematic view of the aligned carbon nanotube-polyethylene composites (ACPCs). (**a**) The perspective view of structure of ACPC 3–8 basic cell; (**b**) and (**c**) The orthographic views of the relaxed structures of ACPC 1–12 and 3–12, respectively; (**d**) The temperature profile of ACPC by non-equilibrium molecular dynamics.

**Figure 2:**
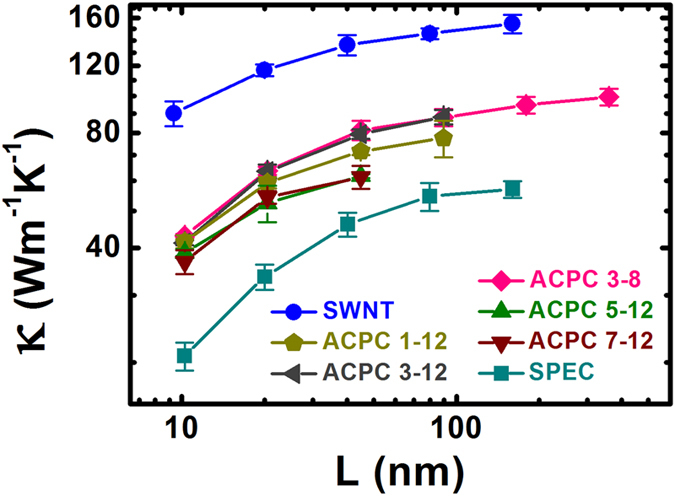
The thermal conductivities (*κ*) of a (10, 10) single-walled carbon nanotube (SWCNT), a suspended polyethylene chain (SPEC), and ACPCs versus the simulation cell’s length. We named ACPC M-N as the structure which have M polyethylene (PE) chains inside the SWCNT and N chains outside the SWCNT. The thermal conductivities of an ACPC M-N are between those of the SWCNT and SPEC. The ACPC 3–8 and ACPC 3–12 have higher thermal conductivities comparing with other ACPC structures. The *κ*_ACPCs_ are not only three orders higher than the bulk PE, but almost twice as large as a SPEC.

**Figure 3:**
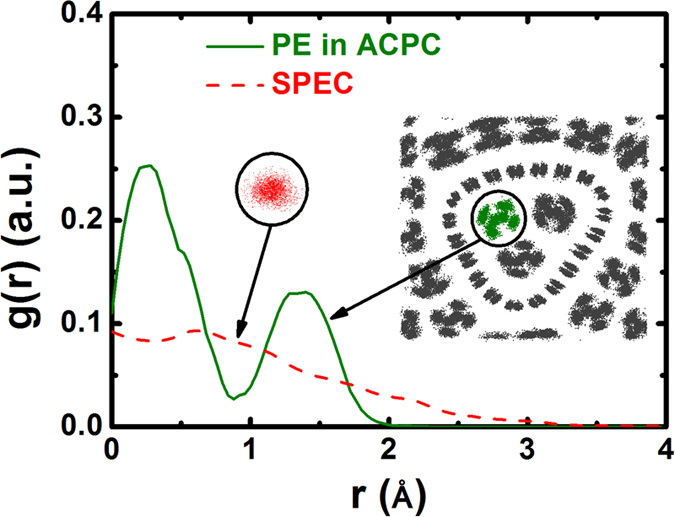
The radial atomic density profiles, g(r), for a SPEC and a PE chain within ACPC 3–12. The red (grey/green) scatters are the superimposed atoms image for a 10 nm SPEC (ACPC 3–12), respectively. The atoms images are established by stacking fifteen frames in a simulation over time.

**Figure 4:**
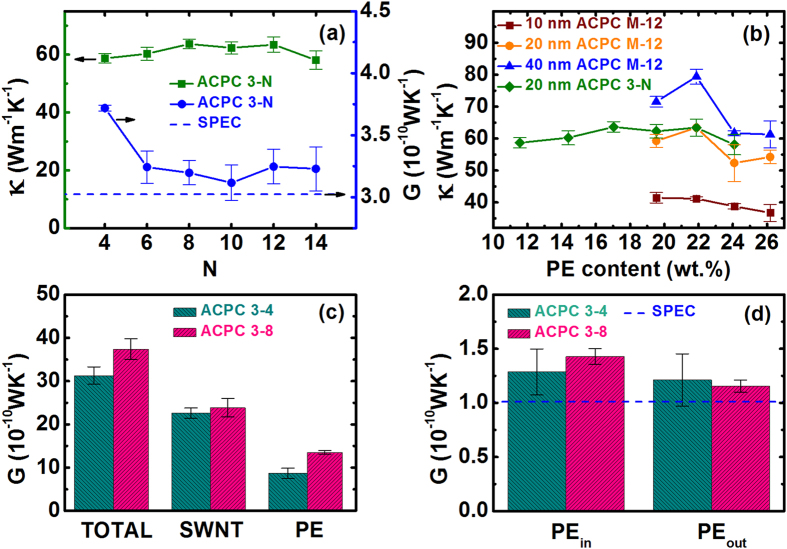
(**a**) The thermal conductivity (*κ*) of ACPC 3-N versus the numbers of chains outside the SWCNT (N). The thermal conductance (G) per PE chain compared to a SPEC at 20 nm length; (**b**) The dependence of *κ*_ACPC_ on PE content for four different ACPC structures. The ACPC 1–12, 3–12, 5–12 and 7–12 correspond to PE contents of 19.53%, 21.88%, 24.09% and 26.18%, respectively. The ACPC 3–4, 3–6, 3–8, 3–10, 3–12 and 3–14 correspond to PE contents of 11.56%, 14.38%, 17.04%, 19.53%, 21.88%, 24.09% and 26.18%, respectively; (**c**) The contributions of SWCNT and PE to the total thermal conductance in an 80 nm length ACPC 3–4 (ACPC 3–8). The PE accounts for 27.8% (ACPC 3–4) and 36.4% (ACPC 3–8), respectively; (**d**) The thermal conductance per PE chain for chains inside (outside) SWCNT in an 80 nm length ACPC 3–4 (ACPC 3–8). The blue dashed line corresponds to the G of an 80 nm SPEC.

Fig. 1(a) shows the typically perspective view of initial positions of ACPC 3–8. The basic cell of the ACPC, which is based on a SWCNT array system, is chosen as the simulation domain to calculate the thermal conductivity for bulk materials by periodic boundary conditions. After the relaxation, the final structures of ACPC 1–12 and 3–12 are shown in Fig. 1(b,c), respectively. Moreover, several different ACPC structures are taken into consideration. We named ACPC M-N as the structure which have M PE chains inside the SWCNT and N chains outside the SWCNT. Fig. 1(d) shows a typical setup and the corresponding temperature profile. The simulation system is divided into 20 or 50 slabs according to the length^40^. The motion equations are integrated by the velocity Verlet algorithm with a time step of 0.2 fs. In calculations of thermal conductivity, it is based on Fourier’s law, 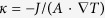, where J is heat flux, A is cross-section area, and T is temperature. The cross-section of (10, 10) SWCNT is defined as a ring with 3.4 Å in thick^41^. Besides, the cross-sectional area of a PE chain is taken as 18 Å^2^ 4. The NEMD method has been detailed in the Ref. 42.

## Additional Information

**How to cite this article**: Liao, Q. *et al.* Extremely High Thermal Conductivity of Aligned Carbon Nanotube-Polyethylene Composites. *Sci. Rep.* **5**, 16543; doi: 10.1038/srep16543 (2015).

## References

[b1] Hu G.-J., Cao B.-Y. & Li Y.-W. Thermal Conduction in a Single Polyethylene Chain Using Molecular Dynamics Simulations. Chinese Phys Lett 31, 086501, 10.1088/0256-307x/31/8/086501 (2014).

[b2] Zhang T., Wu X. & Luo T. Polymer Nanofibers with Outstanding Thermal Conductivity and Thermal Stability: Fundamental Linkage between Molecular Characteristics and Macroscopic Thermal Properties. J Phys Chem C, 21148–21159, 10.1021/jp5051639 (2014).

[b3] Hall J. F. History and Bibliography of Polymeric Insulators for Outdoor Applications. IEEE T Power Deliver 8, 376–385, 10.1109/61.180359 (1993).

[b4] Henry A. & Chen G. High Thermal Conductivity of Single Polyethylene Chains Using Molecular Dynamics Simulations. Phys Rev Lett 101, 235502, 10.1103/PhysRevLett.101.235502 (2008). 19113566

[b5] Henry A. & Chen G. Anomalous heat conduction in polyethylene chains: Theory and molecular dynamics simulations. Phys Rev B 79, 144305, 10.1103/PhysRevB.79.144305 (2009).

[b6] Shen S., Henry A., Tong J., Zheng R. & Chen G. Polyethylene nanofibres with very high thermal conductivities. Nat Nano 5, 251–255, 10.1038/nnano.2010.27 (2010). 20208547

[b7] Singh V. *et al.* High thermal conductivity of chain-oriented amorphous polythiophene. Nat Nanotechnol 9, 384–390, 10.1038/nnano.2014.44 (2014). 24681778

[b8] Huang X., Liu G. & Wang X. New secrets of spider silk: exceptionally high thermal conductivity and its abnormal change under stretching. Adv Mater 24, 1482–1486, 10.1002/adma.201104668 (2012). 22388863

[b9] Loomis J. *et al.* Continuous fabrication platform for highly aligned polymer films. Technology 2, 189–199, 10.1142/S2339547814500216 (2014).

[b10] Iijima S. Helical microtubules of graphitic carbon. Nature 354, 10.1038/354056a0 (1991).

[b11] Yang N., Xu X., Zhang G. & Li B. Thermal transport in nanostructures. AIP Adv 2, 041410, 10.1063/1.4773462 (2012).

[b12] Kim P., Shi L., Majumdar A. & McEuen P. L. Thermal transport measurements of individual multiwalled nanotubes. Phys Rev Lett 87, 215502, 10.1103/PhysRevLett.87.215502 (2001). 11736348

[b13] Yu K. *et al.* Semiconducting Polymers with Nanocrystallites Interconnected via Boron-Doped Carbon Nanotubes. Nano Lett, 7100–7106, 10.1021/nl503574h (2014). 25372930

[b14] Wei C., Srivastava D. & Cho K. Thermal expansion and diffusion coefficients of carbon nanotube-polymer composites. Nano Lett 2, 647–650, 10.1021/nl025554+ (2002).

[b15] Luo C. *et al.* Flexible carbon nanotube− polymer composite films with high conductivity and superhydrophobicity made by solution process. Nano Lett 8, 4454–4458, 10.1021/nl802411d (2008). 19367804

[b16] Vega J. F. *et al.* Rheology, Processing, Tensile Properties, and Crystallization of Polyethylene/Carbon Nanotube Nanocomposites. Macromolecules 42, 4719–4727, 10.1021/ma900645f (2009).

[b17] Haggenmueller R., Fischer J. E. & Winey K. I. S.ingle wall carbon nanotube/polyethylene nanocomposites: nucleating and templating polyethylene crystallites. Macromolecules 39, 2964–2971, 10.1021/ma0527698 (2006).

[b18] Haggenmueller R., Guthy C., Lukes J. R., Fischer J. E. & Winey K. I. S.ingle wall carbon nanotube/polyethylene nanocomposites: thermal and electrical conductivity. Macromolecules 40, 2417–2421, 10.1021/ma0615046 (2007).

[b19] Minus M. L., Chae H. G. & Kumar S. Polyethylene crystallization nucleated by carbon nanotubes under shear. ACS Appl Mater Inter 4, 326–330, 10.1021/am2013757 (2012). 22148325

[b20] Marconnet A. M., Yamamoto N., Panzer M. A., Wardle B. L. & Goodson K. E. Thermal conduction in aligned carbon nanotube–polymer nanocomposites with high packing density. ACS nano 5, 4818–4825, 10.1021/nn200847u (2011). 21598962

[b21] Moniruzzaman M. & Winey K. I. Polymer nanocomposites containing carbon nanotubes. Macromolecules 39, 5194–5205, 10.1021/ma060733p (2006).

[b22] Xu Y., Ray G. & Abdel-Magid B. Thermal behavior of single-walled carbon nanotube polymer–matrix composites. Compos Part A: Appl S 37, 114–121, 10.1016/j.compositesa.2005.04.009 (2006).

[b23] Huxtable S. T. *et al.* Interfacial heat flow in carbon nanotube suspensions. Nat Mater 2, 731–734, 10.1038/nmat996 (2003). 14556001

[b24] Guthy C., Du F., Brand S., Winey K. I. & Fischer J. E. Thermal conductivity of single-walled carbon nanotube/PMMA nanocomposites. J Heat Trans 129, 1096–1099, 10.1115/1.2737484 (2007).

[b25] Hong W.-T. & Tai N.-H. Investigations on the thermal conductivity of composites reinforced with carbon nanotubes. Diam Relat Mater 17, 1577–1581, 10.1016/j.diamond.2008.03.037 (2008).

[b26] Wang M., Galpaya D., Lai Z. B., Xu Y. & Yan C. Surface functionalization on the thermal conductivity of graphene–polymer nanocomposites. Inter J Smart Nano Mater 5, 123–132, 10.1080/19475411.2014.904828 (2014).

[b27] Clancy T. C. & Gates T. S. Modeling of interfacial modification effects on thermal conductivity of carbon nanotube composites. *Polymer* 47, 5990–5996, 10.1016/j.polymer.2006.05.062 (2006).

[b28] Padgett C. W. & Brenner D. W. Influence of chemisorption on the thermal conductivity of single-wall carbon nanotubes. Nano Lett 4, 1051–1053, 10.1021/nl049645d (2004).

[b29] Lukes J. R. & Zhong H. Thermal Conductivity of Individual Single-Wall Carbon Nanotubes. J Heat Trans 129, 705, 10.1115/1.2717242 (2007).

[b30] Moreland J. F. The Disparate Thermal Conductivity Of Carbon Nanotubes And Diamond Nanowires Studied by Atomistic Simulation. Microscale Therrm Eng 8, 61–69, 10.1080/10893950490272939 (2004).

[b31] Gordiz K. & Allaei Mehdi Vaez, S. Thermal rectification in pristine-hydrogenated carbon nanotube junction: A molecular dynamics study. J Appl Phys 115, 163512, 10.1063/1.4873124 (2014).

[b32] Zhang T. & Luo T. Morphology-influenced thermal conductivity of polyethylene single chains and crystalline fibers. J Appl Phys 112, 094304, 10.1063/1.4759293 (2012).

[b33] Yang N., Zhang G. & Li B. Violation of Fourier’s law and anomalous heat diffusion in silicon nanowires. Nano Today 5, 85–90, 10.1016/j.nantod.2010.02.002 (2010).

[b34] Plimpton S. Fast parallel algorithms for short-range molecular dynamics. J Comput Phys 117, 1–19, 10.1006/jcph.1995.1039 (1995).

[b35] Tuzun R. E., Noid D. W., Sumpter B. G. & Merkle R. C. Dynamics of Fluid Flow Inside Carbon Nanotubes. Nanotechnology 7, 241, 10.1088/0957-4484/7/3/012 (1996).

[b36] Quo Y., Karasawa N. & Goddard W. A. Prediction of fullerene packing in C60 and C70 crystals. Nature 351, 464–467, 10.1038/351464a0 (1991).

[b37] Yang N., Zhang G. & Li B. Carbon nanocone: A promising thermal rectifier. Appl Phys Lett 93, 243111, 10.1063/1.3049603 (2008).

[b38] Brenner D. W. Empirical Potential for Hydrocarbons for Use in Simulating the Chemical Vapor Deposition of Diamond Films. Phys Rev B 42, 9458–9471, 10.1103/PhysRevB.42.9458 (1990). 9995183

[b39] Alvarez F. X., Jou D. & Sellitto A. Phonon hydrodynamics and phonon-boundary scattering in nanosystems. J Appl Phys 105, 014317, 10.1063/1.3056136 (2009).

[b40] Liu J. & Yang R. Length-dependent thermal conductivity of single extended polymer chains. Phys Rev B 86, 104307, 10.1103/PhysRevB.86.104307(2012).

[b41] Yao Z., Wang J.-S., Li B. & Liu G.-R. Thermal conduction of carbon nanotubes using molecular dynamics. Phys Rev B 71, 085417, 10.1103/PhysRevB.71.085417 (2005).

[b42] Yang N., Zhang G. & Li B. Ultralow thermal conductivity of isotope-doped silicon nanowires. Nano Lett 8, 276–280, 10.1021/nl0725998 (2008). 18095735

